# Boric Acid Inhibits Germination and Colonization of *Saprolegnia* Spores *In Vitro* and *In Vivo*

**DOI:** 10.1371/journal.pone.0091878

**Published:** 2014-04-03

**Authors:** Shimaa E. Ali, Even Thoen, Øystein Evensen, Ida Skaar

**Affiliations:** 1 Norwegian Veterinary Institute, Oslo, Norway; 2 Norwegian School of Veterinary Science, Oslo, Norway; Louisiana State University, United States of America

## Abstract

*Saprolegnia* infections cause severe economic losses among freshwater fish and their eggs. The banning of malachite green increased the demand for finding effective alternative treatments to control the disease. In the present study, we investigated the ability of boric acid to control saprolegniosis in salmon eggs and yolk sac fry. Under *in vitro* conditions, boric acid was able to decrease *Saprolegnia* spore activity and mycelial growth in all tested concentrations above 0.2 g/L, while complete inhibition of germination and growth was observed at a concentration of 0.8 g/L. In *in vivo* experiments using Atlantic salmon eyed eggs, saprolegniosis was controlled by boric acid at concentrations ranging from 0.2–1.4 g/L during continuous exposure, and at 1.0–4.0 g/L during intermittent exposure. The same effect was observed on salmon yolk sac fry exposed continuously to 0.5 g/L boric acid during the natural outbreak of saprolegniosis. During the experiments no negative impact with regard to hatchability and viability was observed in either eggs or fry, which indicate safety of use at all tested concentrations. The high hatchability and survival rates recorded following the *in vivo* testing suggest that boric acid is a candidate for prophylaxis and control of saprolegniosis.

## Introduction

Aquatic diseases caused by species in genus *Saprolegnia* in farmed freshwater fish and their eggs constitute a major economic problem especially in salmonid hatcheries [1]–[3]. *Saprolegnia* species are ubiquitous in natural water supplying fish hatcheries and have proven difficult to eliminate by filtering and physical treatments (UV, ozone). Several *Saprolegnia* species that cause more than 90% mortality in fish and eggs have been isolated [4]–[6]. In the past this problem was solved with the extremely effective fungicide malachite green (MG) [7], but due to suspected teratogenicity [8] it was banned for use in fish intended for human consumption [9]. The method most often used after the banning of MG involves treatment with formalin-based products. However, formalin is also banned in some countries [10], and several countries are expected to follow, European Union (EU) included. Bronopol is an alternative treatment available in the market [7], but is of inferior effect compared to malachite green. Attempts have been made to find alternatives to MG [5], [11]–[13], but still no comparably effective alternatives have been found.

Boric acid (BA) and its salts, borates, have been used in medicine as bactericides, fungicides, and antiseptics since the 1860s [14]. BA was originally registered as a pesticide in the U.S. in 1948; there are currently 189 registered pesticide products on the market containing BA or one of its sodium salts as an active ingredient [15]. It is a recommended treatment for some fungal infections in humans [16] and plants [17]. However, no information is available concerning the antimicrobial activity of borates against fungi or oomycetes pathogenic to fish. Therefore, we aimed to explore its potential as a treatment against *Saprolegnia* infections of eggs and yolk sac fry. The high survival rate following continuous and intermittent exposure of eyed eggs and yolk sac fry to BA is an indication of its safety for use. However, more investigations regarding BA environmental impact should be performed.

## Materials and Methods

### Ethics Statement

According to the Norwegian regulations on animal experimentation, no special permissions were required for eyed eggs or yolk sac fry experiments. The legislation applies from the time larvae start actively to feed, not before. In all experiments, embryos were manipulated and sacrificed prior to yolk sac absorption (maximum three weeks following hatching).

### Chemicals

Boric acid H_3_BO_3_, M 61.83 g/mol (Merck) was used as a source for borate to test its effect on the germination of *Saprolegnia* spores and the growth of *Saprolegnia* hyphae (*in vitro*). BA was also tested for control of *Saprolegnia* infection of eyed salmon eggs and yolk sac fry (*in vivo*) and its safety *in vivo* was also assessed.

### *Saprolegnia* strains used for *in vitro* testing

Three strains of *Saprolegnia* sp. were used to evaluate the *in vitro* effect of the BA on germination and colonization of *Saprolegnia* spores and also anti-growth effects on hyphae/mycelium. Two strains (VIO 2736, VIO 5708) were *S. parasitica* isolated from Atlantic salmon (*Salmo salar* L.), the third strain was *S. diclina* (VIO2739) isolated from infected Atlantic salmon eggs.

### Concentrations of boric acid (BA) tested

BA was diluted to desired concentration in Sterilized Aquarium Water (SAW). It was tested initially at 0.01, 0.1 and 1.0 g/L to find out the preliminary minimum inhibitory concentration (MIC) intervals *in vitro*. Once the concentration range able to inhibit *Saprolegnia* spore germination and arrest hyphal growth was determined, the test intervals were narrowed down and concentrations between 0.1 and 1.0 g/L were tested at incremental increase of 0.1 g/L.

### Effect of boric acid on the germination and colonization of *Saprolegnia* spores

Sesame seeds were autoclaved at 121°C for 20 min and cooled. *Saprolegnia* zoospores were produced according to the method described previously by Stueland et al. (2005). 400 µl of *Saprolegnia* spore suspension (1.0×10^4^ spores/L) and 400 µl of each tested BA concentration were incubated with sesame seeds in 24-well, flat-bottom plates (COSTAR). Sterilized aquarium water (SAW) was used as a non-treated control and bronopol (Pyceze vet. 500 g bronopol/L) was used as a positive control. Each BA concentration and controls were tested in triplicates and incubated at 20°C. 24 hours later, plates were examined microscopically. The germination and colonization of *Saprolegnia* spores on sesame seeds were graded as follows:

Grade 0: no germination, no growing mycelia on the sesame seeds

Grade 1: germinating spores, no growing mycelia on the sesame seeds

Grade 2: germinating spores, very few growing mycelia on the sesame seeds

Grade 3: germinating spores, profuse growing mycelia on the sesame seeds

In addition to the qualitative evaluation based on seeds system, the inhibitory effect of BA on the germination of encysted *Saprolegnia* spores was also quantified following 6 h incubation with the treatment. Cysts in water were used as a non-treated control. 1% of GY broth was added to all tested concentrations and controls to enhance the germination. The numbers of germinating *Saprolegnia* cysts were counted directly under the microscope and the mean was calculated based on ten well replicates per concentration.

### Effect of boric acid on the growth of *Saprolegnia* mycelium

400 µl of *Saprolegnia* spore suspensions were incubated with sterile sesame seeds using flat-bottom microwell plates for 12 h at 20°C. The plates were observed microscopically to confirm the attachment of the spores to the sesame seeds and the presence of the early growing mycelia. BA was added at 12 h post infection of the sesame seeds at the same concentrations mentioned above to all tested plates except controls. Plates were incubated further at 20°C. SAW was used as non-treated control and bronopol as positive control. Plates were inspected microscopically after 24 h for advanced mycelial growth. Mycelial growth on the sesame seeds was graded as 0 for complete inhibition of the growth, and grade 1 for slight reduction on the growth or the presence of abundance of the growing mycelium on the sesame seeds.

For the quantitative assessment of the inhibitory effects of BA on the hyphae growth, the method described by Beakes and Gay (1980) [18] was applied. Briefly, BA was dissolved in sterilized distal water and incorporated into molten agar glucose yeast (GY) held at 45°C to give final concentrations from 0.1–1.0 g/L (ten tested concentrations). Each BA concentration and the non-treated controls were tested in triplicates. All plates were inoculated with agar plugs (2 mm) obtained from 24 h growing *Saprolegnia* culture and incubated at 20°C. The colony diameters were measured and the average radial colony growth was calculated following 72 h incubation.

### Effect of boric acid on the development and sporulation of *Saprolegnia* sporangia

Sesame seeds with 24 h growing hyphae were incubated with different boric acid concentrations (0.1–1.0 g/L) and in SAW as a non-treated control. The numbers of developed *Saprolegnia* sporangia were counted every 2 h following the induction of sporulation. The mean sporangia numbers were calculated based on 3 replicates per concentration.

### Effect of boric acid on early developing *Saprolegnia* zoosporangia

Sesame seeds carrying *Saprolegnia* mycelium with zoosporangia were examined microscopically after their exposure to 1 g/L of BA treatments for 12 and 24 h.

### Viability of treated *Saprolegnia* spores and zoosporangia post treatment

*Saprolegnia* spores and zoosporangia were treated with boric acid at 1 g/L (diluted in SAW) for various time periods, 12, 24, 48, 72 and 96 h. At indicated time points BA was replaced with SAW. The ability of treated spores to germinate and colonize sesame seeds was recorded 24 h following the treatment removal. Treated zoosporangia were also examined for their ability to release spore. Spores and zoosporangia in SAW were used as a non-treated control. Results were confirmed with fluorescent microscopy using SYTO 9 dye (Invitrogen) 5 mM solution in DMSO.

### *Saprolegnia* strains used for *in vivo* testing

Two strains of *Saprolegnia* spp. (*S. diclina* VIO 2739 and *S. parasitica* VIO 2741) were used for *in vivo* testing. Both strains had previously been proved to be pathogenic for salmon eggs [19]. Cysts were produced according to the method described earlier [5]. The cysts were counted using a haemocytometer (Bürker türk). The cyst suspensions were adjusted by dilution to obtain the required density 1.0×10^4^ spores/L [19].

### Origin and treatment of salmon eggs

Atlantic salmon eyed eggs (AquaGen strain) with the following criteria were used: 385 degrees days post fertilization at shipment. All batches of eggs were formalin treated as standard procedure during incubation, and disinfected with buffodine (1∶100, 10 min) before shipment. Temperature range during incubation was 2.4–8.0°C; average: 4.2°C. The parent population had been screened for infection with salmon pancreas disease virus (causing pancreas disease), infectious salmon anemia virus (causing ISA) and for *Renibacterium salmoninarum* (causing bacterial kidney disease). After arrival to the laboratory aquaria at the Norwegian Veterinary Institute/Norwegian School of Veterinary Science, eggs were transferred to an incubator which was supplied with continuous water flow (0.8 L/min per liter of eggs) with water temperature ranging from 5 to 7°C. To avoid mortality from transportation damage, eggs were acclimatized for 3 days before they were used in the experiment.

### Preparation of infected dead eggs (source of infection)

Groups of live eyed salmon eggs were killed by immersion for one min in water bath at temperature of 60°C [19]. Dead eggs were incubated in 24-well microwell plates with *Saprolegnia* spore suspensions, 1.0×10^4^ spores/L at 15°C for 48 h. Incubated eggs were examined microscopically for the presence of *Saprolegnia* hyphae.

Three different challenge experiments were performed on salmon eggs and one on salmonid yolk sac fry obtained from the hatched eggs as described below.

### Effect of continuous exposure to BA

In this experiment, we aimed to test the safety of continuous exposure of live eggs to different concentration of BA, and to test the activity of BA with regard to its ability to control the spread of *Saprolegnia* sp. infection between eyed salmon eggs. The setup allowed us to determine the optimal BA concentration which is safe for eyed eggs and at the same time able to control the spread of infection between eggs.

About 730 live eggs were used in this experiment. They were divided in to 9 main groups and tested in seven different BA concentrations plus two controls as described below. Each group was subdivided into 3 replicates of 27±2 eggs. Live eggs were challenged by co-incubation with infected dead ones (three infected dead eggs/replicate) [19]. Eggs were placed in a container to form an even layer (1 egg layer) and to be in close contact with the source of infection. To control the incubation temperature, all the containers with different BA concentrations tested and the controls were kept in a water bath with continuous water flow. Each container was supplied with an aerator. Water temperature during the experimental period ranged from 4–7°C, the pH ranged from 6.8–7.0.

In all treated groups, eggs were exposed to BA continuously until most of the eggs were hatched. BA treatment was tested at the following concentrations; 0.2, 0.4, 0.6, 0.8, 1.0, 1.2, 1.4 g/L. Eggs incubated in aquarium water were used as non-treated negative controls and those exposed to bronopol served as positive controls. Eggs were examined every day for attachment between the infected dead eggs and live ones, presence of any dead eggs, time of egg hatching, hatching rate and the presence of any yolk sac fry abnormalities, as described by Thoen et al. (2011).

### Intermittent exposure to BA

This experiment was designed to test the effect of intermittent exposure to BA with regard to its ability to control the spread of *Saprolegnia* sp. infection between eyed salmon eggs. The experimental design was as described in experiment 1 with the same number of eggs per replicate (N = 27±2) and the number of infected eggs introduced per replicate (3 per 27 eggs). The only difference was that the eggs were exposed to higher BA concentrations (1, 2, 3, 4 g/L) for 4 h once a day over the course of the experiment which lasted for 14 days in total. After 4 h of treatment the flow of aquarium water was resumed. The same treatment was applied 24 h later and every day up to 14 days. The time between treatments was the same throughout the experiment. The number of dead eggs, presence or absence of infection and hatching rates were recorded over the duration of the experiment.

Infected dead eggs that were used as source of infection in the BA treated groups and the non-treated control were examined microscopically to observe the effect of BA treatment on the growing mycelia.

### Protection against infection of dead eggs

This experiment was designed to investigate the ability of BA to protect dead eggs from becoming infected with *Saprolegnia* spores. The rationale is that dead eggs are the first to get infected under natural conditions and act as a source of infection for live eggs.

Five egg groups were used, each group divided equally into 3 smaller sub-groups. 25 live eggs plus 5 dead ones were placed in each of the sub-groups (90 eggs per group). Dead eggs were prepared as described above. In all groups, eggs were supplied with continuous slow water flow of 1 L/min. The aquarium water at the facilities of the Norwegian Veterinary Institute/Norwegian School of Veterinary Science contains *Saprolegnia* zoospores [20] and was used as source of infection. In addition, to ensure presence of *Saprolegnia* spores in the in-coming water, growing mycelia from *S. diclina* (VIO 2739) and *S. parasitica* (VIO 2741) were packed in a piece of gauze and fixed inside the main tubes that supplied the water to all containers. Treatment with BA was carried out as follows. The water flow was stopped and each group, except the non-treated control group, was treated with BA at concentrations of 1, 2, 3, and 4 g/L. The treatment period was 4 hours/day after which water flow was resumed. Dead eggs were inspected daily over 14 days for their colonization with *Saprolegnia* spores and the presence of growing hypha. Number of dead eggs per group and hatching rates were recorded.

### Protection against natural infection in yolk sac fry

This experiment was designed to test the safety of continuous exposure of salmon yolk sac fry to a low concentration of BA (0.5 g/L). We also tested to what extent BA will protect Atlantic salmon yolk sac fry from becoming infected by *Saprolegnia* spores during a natural outbreak of saprolegniosis in the aquarium.

About 330 live, yolk-sac fry of Atlantic salmon obtained from treated hatched eggs were used in this experiment. They were divided into two equal groups. Group 1 was kept in aquarium water naturally containing virulent *Saprolegnia* spores [20]. Group 2 was put on continuous treatment with BA at a concentration of 0.5 g/L. The containers were supplied with air through air pumps. Yolk sac fry were observed daily and the numbers of dead yolk sac fry per day were recorded in both groups. To confirm whether the high mortality rates were due to *Saprolegnia* infection, live and freshly dead yolk sac fry were collected and examined from both groups by the end of the experiment. They were examined microscopically and incubated on glucose yeast agar with antibiotics. Plates were kept at 20°C for 24 h and examined for the presence of *Saprolegnia* mycelia.

### Statistical Analysis

Statistical analyses were performed with the help of GraphPad Prism 5.0 using chi-square test. The level of significance was set at p<0.05.

## Results

**Figure 1:**
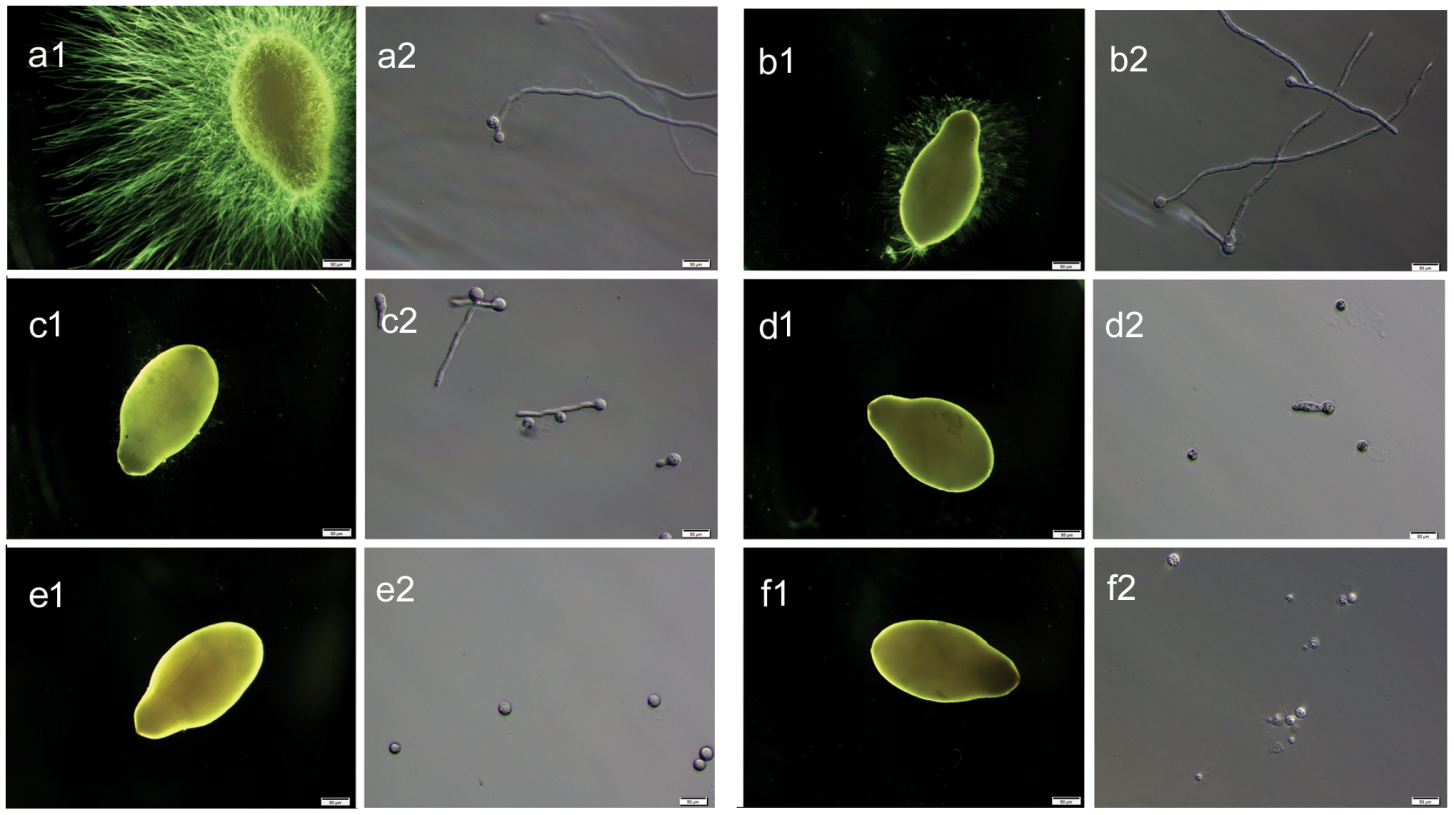
*In vitro* effect of boric acid on the germination and colonization of *Saprolegnia* spores. Effect of boric acid on the germination and colonization of *Saprolegnia* spores following 48 hours incubation. (**a1 and a2**) Grade 3: Profuse *Saprolegnia* hyphal growth on sesame seeds in the non- treated control group (water). (**b1 and b2**) Grade 3: germinating spores with mycelial growth on the sesame seeds (boric acid <0.2 g/L). (**c1 and c2**) Grade 2: reduced germination rate and minimal mycelial growth on the sesame seeds (boric acid 0.2–0.4 g/L). (**d1 and d2**) Grade 1: germinating spores without growing mycelia on the sesame seeds (boric acid 0.5–0.7 g/L). (**e1 and e2**) Grade 0: no germinating spores, no growing mycelia on the sesame seeds (boric acid ≥0.8 g/L). (**f1 and f2**) Grade 0: positive control group (bronopol), no spore germination with absence of the growing mycelia on sesame seeds.

**Figure 2:**
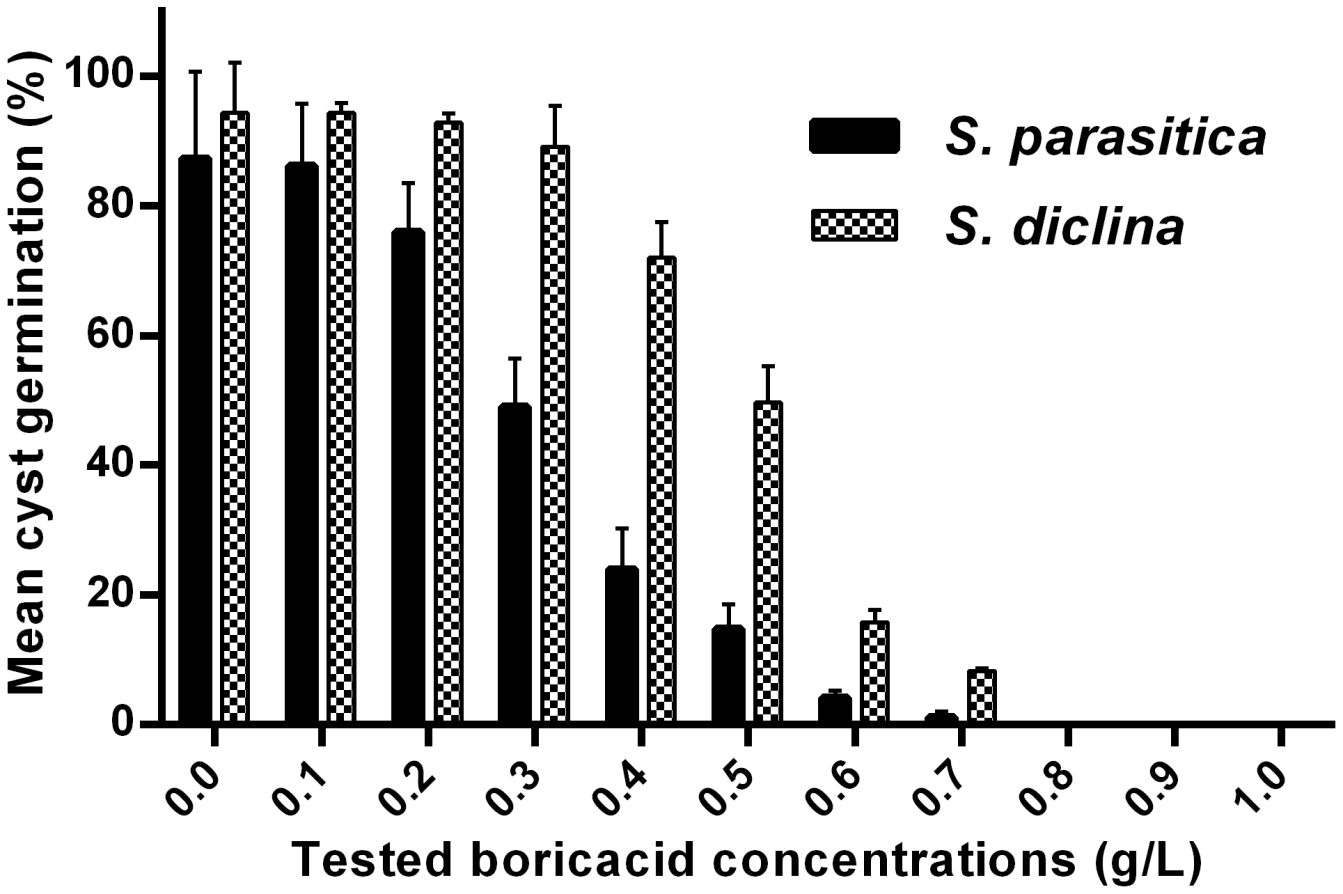
Effect of boric acid on *Saprolegnia* cyst germination. The mean number of germinating *Saprolegnia* cysts counted at 200× magnification 6 h following incubation with different boric acid concentrations.

**Figure 3:**
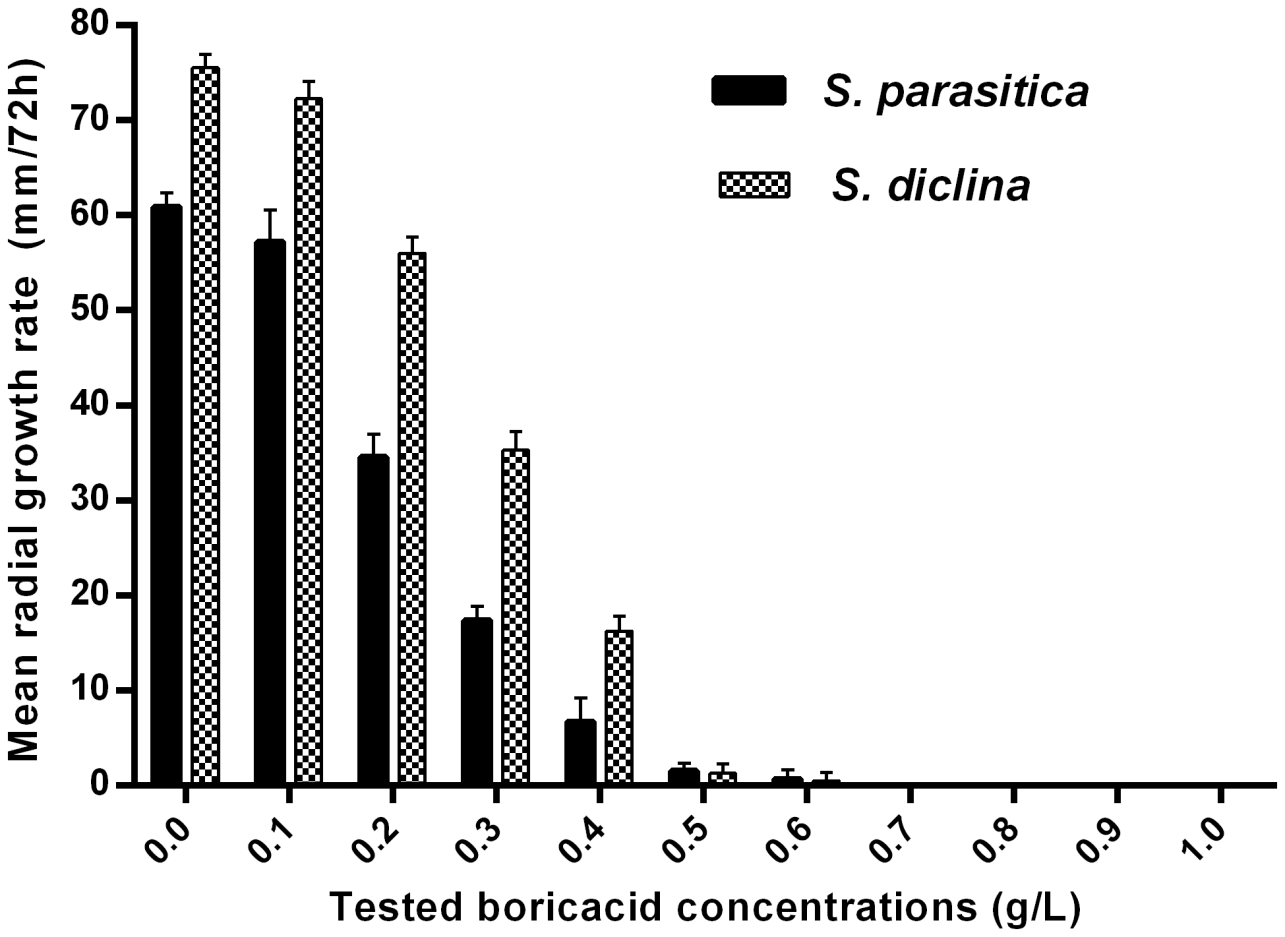
Effect of boric acid on *Saprolegnia* radial growth rate. The mean radial growth rate of two *Saprolegnia* sp. following 72 h incubation on glucose yeast agar mixed with different boric acid concentrations.

Boric acid inhibits germination and colonization of *Saprolegnia* spores *in vitro* and has a static effect on mycelium growth. Our first approach was to test the ability of boric acid to influence germination and colonization of *Saprolegnia* spores *in vitro*. *Saprolegnia* spores germinated and colonized sesame seeds in all groups at BA concentrations below 0.2 g/L, however, the growth rate was slow compared to the non-treated control even at this low concentration. When BA concentrations of 0.8 g/L and above were used, inoculated *Saprolegnia* spores did not germinate (Table 1 & Fig. 1 and 2). We also tested the effect on mycelial growth and at concentrations above 0.6 g/L, BA arrests mycelia and no further growth is seen (Table 2 & Fig. 3).

**Table 1:** Effect of boric acid on the germination and colonization of *Saprolegnia* spores.

Tested BA concentrations	<0.2 g/L	0.2–0.4 g/L	0.5–0.7 g/L	≥8 g/L
Grades*	3	2	1	0

Grade 0: no germination, no growing mycelia on the sesame seeds.

Grade 1: germinating spores, no growing mycelia on the sesame seeds.

Grade 2: germinating spores, very few growing mycelia on the sesame seeds.

Grade 3: germinating spores, profuse growing mycelia on the sesame seeds.

* Average of 3 replicates.

**Table 2:** Effect of different boric acid concentrations on the radial growth rate of two *Saprolegnia* sp. (*S. parasitica* and *S. diclina*).

Tested BA concentrations in g/L	*S. parasitica*		*S. diclina*	
	Mean radial growth in mm*	Colony growth inhibition (%)	Mean radial growth in mm*	Colony growth inhibition (%)
0.0	61	0	76	0
0.1	57	7	72	5
0.2	35	43	56	26
0.3	17	72	35	54
0.4	7	89	16	79
0.5	2	97	1	99
0.6	1	98	1	99
0.7	0	100	0	100
0.8	0	100	0	100
0.9	0	100	0	100
1.0	0	100	0	100

*Based on 3 replicate colonies.

### Boric acid arrests formation and development of *Saprolegnia* zoosporangia

**Figure 4:**
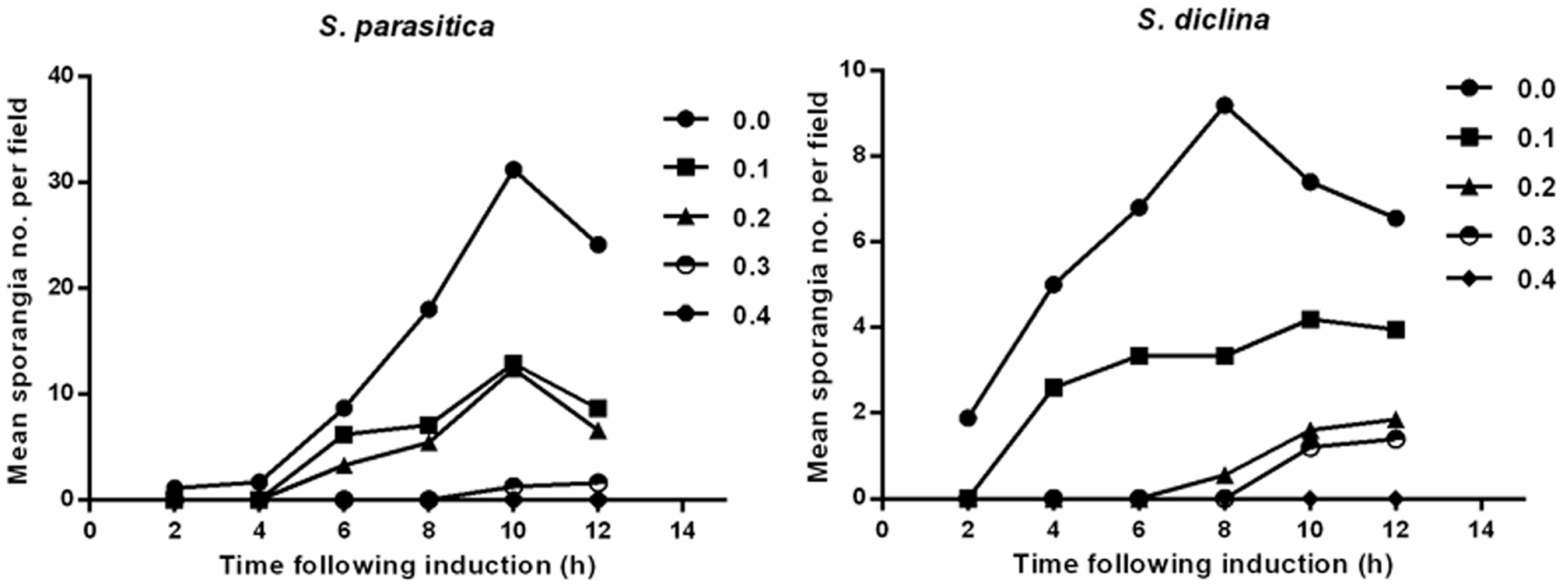
Effect of boric acid on the development and formation of *Saprolegnia* sporangia. The mean number of developed sporangia of two *Saprolegnia* sp. counted at 200× magnification following incubation with different boric acid concentrations.

**Figure 5:**
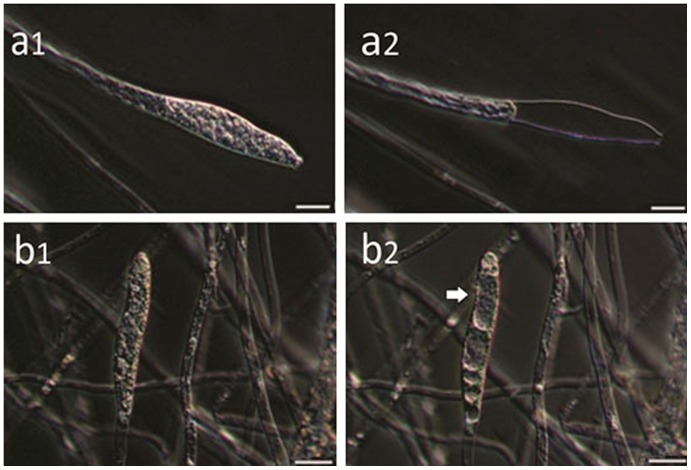
*In vitro* effect of boric acid on the development of *Saprolegnia* zoosporangia. *Saprolegnia* zoosporangia in the non-treated control before (**a1**) and after (**a2**) spore release. *Saprolegnia* zoosporangia before (**b1**) and after 24 hours exposure to boric acid treatment (**b2**). Notice the degenerative changes within the zoosporangia and absence of spore production and release (b2; arrow).

We then went on to assess the impact of boric acid on the development of sporangia and the maturation of young *Saprolegnia* zoosporangia. We found that boric acid arrest sporangia formation at 0.4 g/L (Fig. 4) and interferes with the production and subsequent release of zoospores at 1.0 g/L. Vacuolation of the entire of zoosporangia was the most prominent sign observed after treatment (Fig. 5).

### Post-treatment spore release is dependent on length of exposure to BA

**Figure 6:**
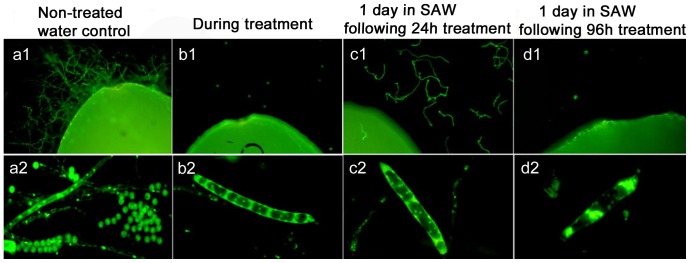
Viability of treated *Saprolegnia* spores and zoosporangia during treatment and post-treatment removal. Fluorescence microscopy showing the viability of boric acid treated *Saprolegnia* spores and zoosporangia with SYTO 9 stain. **a1**) Colonization is seen on control seed. **a2**) Normal zoosporangium sporulating. **b1**) Non-colonized seed during treatment. **b2**) Effect on zoosporangium during treatment. **c1** and **c2**) Spores and zoosporangium were treated for 24 h with BA and left in sterilized aquarium water (SAW) for another 24 h after treatment was terminated. Germlings can be seen surrounding the seeds in c1. **c2**) shows a close-up of zoosporangium treated as in c1. **d1** and **d2**) Spores and zoosporangium were treated for 96 h in BA and then left in SAW for 24 h after treatment was terminated. There is no colonization on seeds after 96 h BA treatment (**d1**) and the zoosporangium (**d2**) appears with condensed staining and a thin wall.

Having shown that BA arrests development of zoosporangia it was our interest to test if treated zoosporangia would release spores after treatment was terminated and we tested different exposure times to BA. Some zoosporangia that were treated with BA for 12 and 24 h showed spore release 24 h after exposure had been terminated (Table 3). In contrast, in samples exposed to BA for 72 and 96 h no spore release was shown (Fig. 6d). Massive spore release was recorded in the non-treated control group (Fig. 6a).

**Table 3:** Viability of treated *Saprolegnia* spores 24 h following removal of boric acid.

Exposure time	Grades (average of 3 replicates)		
	During treatment 1 g/L	24 h following treatment removal	Non-treated control
12 h	0	1	3
24 h	0	1	3
48 h	0	1	3
72 h	0	0	3
96 h	0	0	3

Grade 0: no germination, no growing mycelia on the sesame seeds.

Grade 1: germinating spores, no growing mycelia on the sesame seeds.

Grade 2: germinating spores, very few growing mycelia on the sesame seeds.

Grade 3: germinating spores, profuse growing mycelia on the sesame seeds.

### Continuous treatment with BA prevents infection of fertilized eggs at concentration above 0.2 g/L

Our next approach was to assess to what extent continuous treatment with BA would prevent infection of fertilized eggs. We used the co-incubation method [19] where live eggs were challenged by introducing killed and experimentally infected dead into the trays. The concentrations used were ranged from 0.2 to 1.4 g/L with an incremental increase of 0.2 g/L between groups (7 in total). Three infection foci were used per replicate.

In the non-treated controls and 3 days after introduction of the infected, dead egg, the hyphae started growing out from infected dead eggs and colonized live surrounding eggs. In the BA treated groups no colonization was observed.

On day 5 post challenge, eggs in the non-treated groups started to coalesce and formed a one-unit appearance of dead eggs, completely covered with *Saprolegnia* mycelium. Still no sign of infection was observed in the treated groups, and no dead eggs were observed. In the group treated with BA at 0.2 g/L, one infection focus was found with slight attachment between two live eggs and infected dead egg. On day 7 post challenge, eggs started to hatch in the non-treated group and some of the treated groups (0.4 and 1.2 g/L). The eggs in the groups treated at concentrations 0.2, 0.6, 0.8, 1.0 and 1.4 g/L hatching started 1 day later.

Results obtained during the second week were similar to what seen during the first week where no infection was recorded in any of the concentrations tested except 0.2 g/L. At this concentration, slight attachment was observed between the egg-shell of the live eggs and the infected dead egg (source of infection). This did not cause the foetus to die as eggs hatched and yolk sac fry swam away from the eggshell.

**Figure 7:**
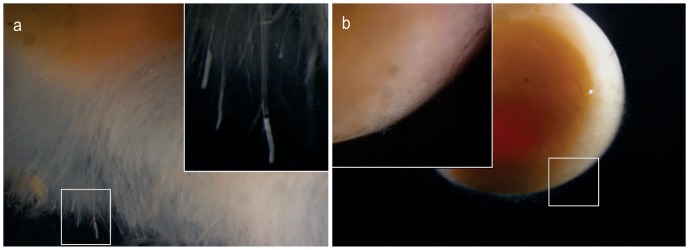
Effect of continuous boric acid treatment on salmon eggs used as infection source. Microscopical examination of infected dead eggs used as a source of infection after the termination of the continuous exposure experiment. Mature *Saprolegnia* zoosporangium (**a**) in the non-treated control group compared to treated one exposed continuously to boric acid treatment (0.6 g/L) (**b**).

**Figure 8:**
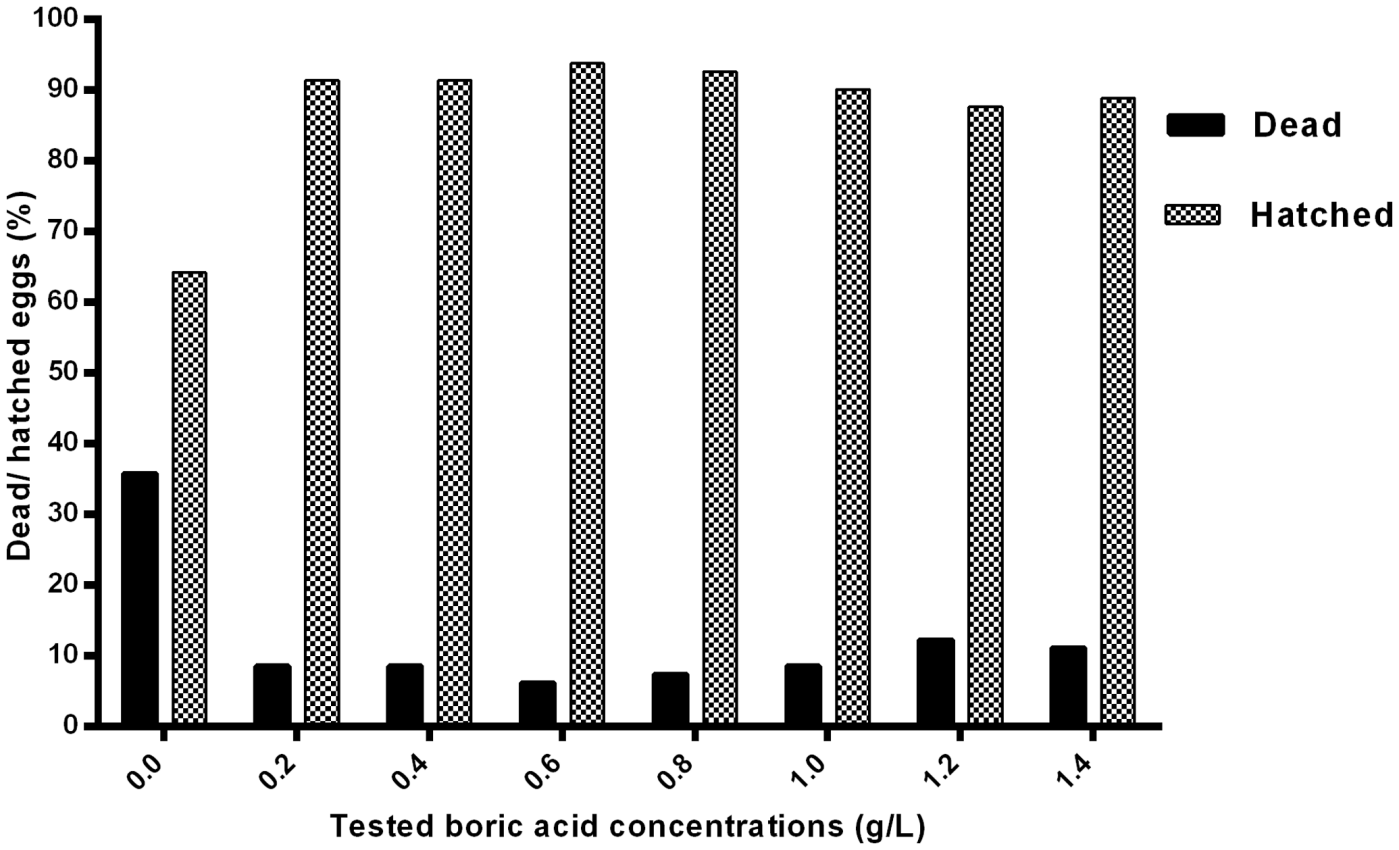
Continuous exposure to boric acid. Mortality and hatching rates in boric acid treated groups and the non-treated control (0.0 g/L) at the end of the first experiment.

At the end of the experiment and in groups treated at BA concentration >0.2 g/L the mycelium covering the eggs that were used as source of infection was scant, while many infected dead eggs were detected in the non-treated control group (Fig. 7). The percent of dead and hatched eggs were recorded after the termination of the experiment (Fig. 8). The bronopol treated group (positive control) showed 100% mortality after 4 h continuous exposure.

### Intermittent exposure with BA prevents the spread of infection to fertilized eggs

**Figure 9:**
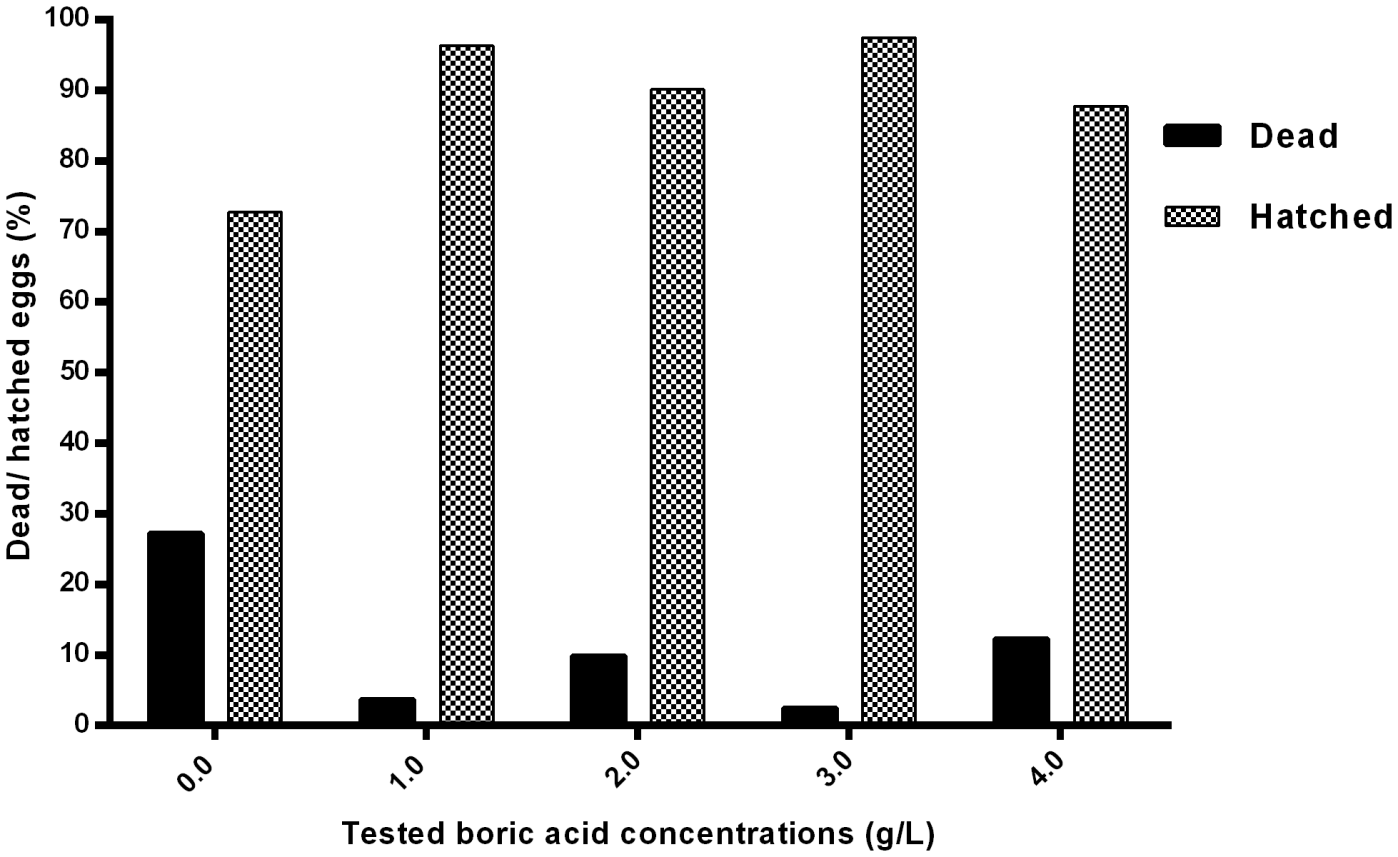
Intermittent exposure to boric acid. Mortality and hatching rates by the end of the experiment in boric acid treated groups and non-treated control (0.0 g/L).

Since continuous treatment with BA prevents infection, we were interested to check out if intermittent treatment would also prevent the spreading of infection from dead eggs to live ones using the same co-incubation method as above. We used BA at concentrations of 1, 2, 3, or 4 g/L and exposure was for 4 h once a day. Many eggs in the non-treated control group died as they were colonized by *Saprolegnia* mycelia. Microscopically, characteristic *Saprolegnia* zoosporangia were observed with release of motile zoospores. No hyphal attachment between infected dead and live eggs were observed at any of the concentrations tested. Dead eggs that were used as a source of infection in all treated groups showed scanty attachment of mycelium. When examined microscopically, only remnants of mycelia mixed with some organic debris from the water were seen. Figure 9 shows the percent of dead and hatched eggs obtained after termination of the experiment.

### Daily treatment with BA protect against *Saprolegnia* infection of dead eggs

**Figure 10:**
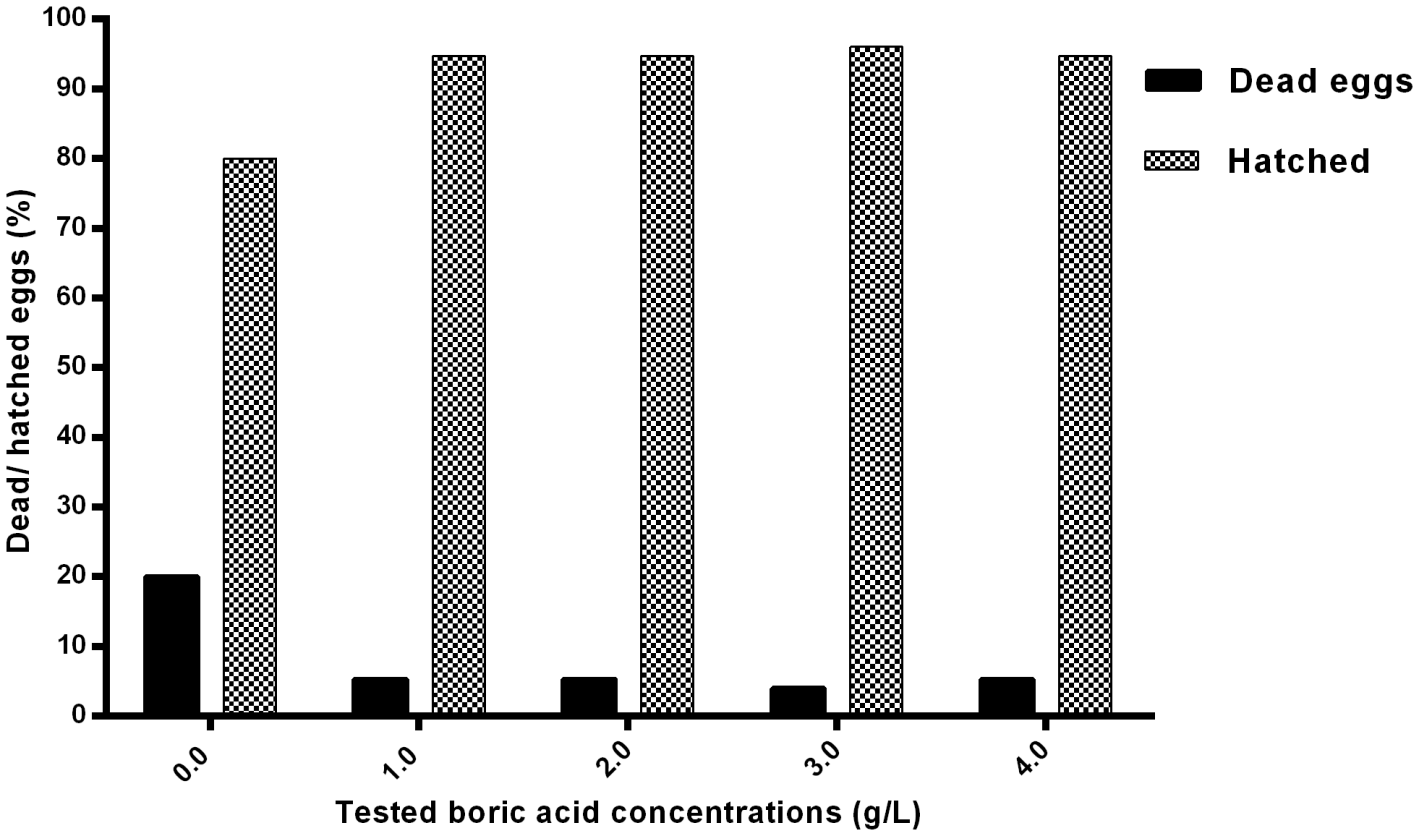
Protection against infection of dead eggs. Mortality and hatching rates in egg groups intermittently exposed to boric acid (third experiment) compared to non-treated control (0.0 g/L).

No hyphal growth was observed on dead eggs in any of the treated groups (1–4 g/L) and subsequently no attachment was found between dead and live eggs. In the water control group, growing *Saprolegnia* mycelia were detected on the dead eggs by the fifth day after challenge. At later stages, the growing mycelia from the dead eggs showed more pronounced growth and penetrated the surrounding live eggs. By the end of the experiment (day 14) the percent of dead and hatched eggs were recorded (Fig. 10).

### Continuous treatment with BA protect yolk sac fry against natural infection

**Figure 11:**
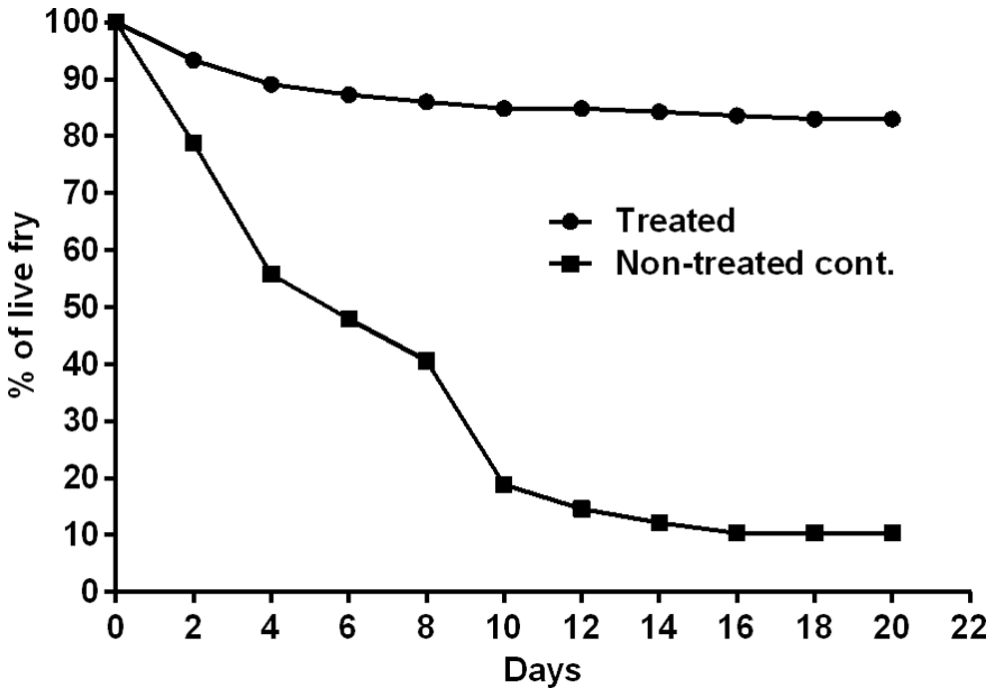
Protection against natural infection in yolk sac fry. Survival curve of treated salmonid yolk sac fry and control during the outbreak of saprolegniosis. Note the significant reduction in mortality among treated group (p<0.001).

Significant reduction (p<0.0001) in yolk sac fry mortality rates (Fig. 11) was observed in the BA treated group compared to the non-treated control. Dead yolk sac fry were completely covered with *Saprolegnia* mycelia. Microscopical examination revealed tufts of white mycelia causing gill obstruction of live and freshly dead yolk sac fry in the non-treated control group. The characteristic *Saprolegnia* zoosporangia were also observed microscopically. Results were confirmed with isolation on GY agar and broth.

In all experiments, the differences between each tested concentration and the non-treated control group were significant (p<0.05).

## Discussion

In this study, we show for the first time that continuous or intermittent treatment with boric acid (BA) inhibits germination and colonization of *Saprolegnia* spores under laboratory conditions. The inhibition ranged from minimal to complete arrest of growth depending on the concentrations tested. We have also shown that BA is efficient at preventing infection of salmon eggs and yolk sac fry during natural and experimental infections. BA is practically nontoxic to fish and aquatic invertebrates [15] and it has a low bioaccumulation potential [15]. Additionally, boron has been identified as a growth stimulator for embryonic rainbow trout, both in the presence and absence of several known essential elements [21]. That effect was observed with very low boron concentration. A similar effect of boron has been observed on zebrafish (*Danio rerio*) embryos [22]. Many reports have demonstrated the antimycotic activity of borate in controlling fungal and yeast infections in humans and plants [16], [17], [23], [24]. Under *in vitro* conditions, the germination and activity of *Saprolegnia* spores were inhibited with BA at 0.8 g/L. Further to this, the germination of *Saprolegni*a spores and the length of the developed germ tube were reduced greatly when BA was applied at lower concentrations (0.5–0.7 g/L). At similar concentrations (above 0.6 g/L), the early developing *Saprolegnia* mycelia on sesame seeds did not show advanced growth and 100% radial growth inhibition was recorded.

We found it particularly interesting that BA controlled the spread of infection from dead, infected eggs over to live, eyed eggs when incubated together. The mycelia that covered the infected dead eggs used as the source of infection did not show expansive growth using an intermittent or continuous BA treatment regime in contrast to non-treated controls. Moreover, following continuous treatment the mycelia that covered dead eggs (used as source of infection) disappeared at the end of the treatment period which would indicate the mycelium being killed. The effect was so distinct that it was difficult to differentiate between dead eggs used as infection source and those that died during the experiment (of natural causes). This is suggesting that continuous exposure to the BA is able to kill *Saprolegnia* hyphae but additional studies are needed to fully substantiate these findings. Taking the other parameters into account (hatching rates, percent of dead eggs after treatment, fry viability), we found that the concentrations that would give an optimal output ranged from 0.6 to 0.8 g/L. We can also conclude that intermittent exposure to the BA also has a very good effect on preventing the spread of infection. According to our results, and from the economical point of view, the best concentration to be used is 1.0 g/L for 4 h/day. All in all this indicates that BA can be a good candidate for controlling *Saprolegnia* infections of eggs and fish.

As we have shown here by light and fluorescent microscopy, BA prevents the development of *Saprolegnia* zoosporangia, consequently no spore release was detected when BA was applied at this stage. BA does not kill *Saprolegnia* spores or hyphae immediately and we base this on the fact that some fully mature zoosporangia showed spore release shortly after BA application. The observation that some spores/zoosporangia treated with BA germinated/exhibited spore release after replacing treatment with SAW indicates fungistatic rather than fungicidal activity of BA at the concentration tested. As regards the effect of BA on *Saprolegnia* mycelium, (infected dead eggs used as a source of infection) the efficacy was positively correlated with the BA concentrations tested. It is of importance that at high concentrations (2.0, 3.0, 4.0 g/L) the hatchability, viability and hatching time were not different from what was found at lower concentrations tested (1.0 g/L). Moreover, no defects, morphological or behavioural abnormalities were observed in newly hatched yolk sac fry. This indicates that BA has a broad therapeutic window and is still safe for use in eggs at such high concentrations.

From microscopic examinations, the characteristic *Saprolegnia* hyphal structure was completely lost in the mycelium on all treated dead eggs used as a source of infection. On the other hand, infected dead eggs in the non-treated control groups showed well-developed *Saprolegnia* zoosporangia with release of motile zoospores. It is widely accepted that dead eggs can easily be colonized by *Saprolegnia* spores and act as a source of infection for live eggs [7], [19], [25]. Continuous treatment is not a preferred option but the observation that intermittent exposure to BA protected dead eggs from being infected with *Saprolegnia* spores opens up for using BA as a prophylactic treatment against saprolegniosis in aquaculture facilities. Additionally, the present study showed that BA served as an efficient treatment to reduce yolk sac fry mortality during a water-borne infection with *Saprolegnia* in the laboratory aquarium which would indicate that BA can also be used for treatment of ongoing infections. Additional studies are needed to elucidate this in more detail. When BA was used at 0.5 g/L, mortalities were reduced from 90% in the untreated water control group to 17% in the treated one (p<0.0001). The low mortalities recorded in the treated group and the absence of the gross abnormalities following long time exposure indicate that BA is safe for used in salmon yolk sac fry at this concentration.

## Conclusion

From the present study we can conclude that BA has a good potential as a prophylactic measure and also a potential as a curative intervention against *Saprolegnia* infection in fertilized eggs and yolk sac fry of Atlantic salmon, and possibly other species. Due to the high hatchability and survival rates recorded after the treatment, BA is considered safe for use in salmon eggs and yolk sac fry. However, further investigations on BA as prophylactic treatment during an outbreak of *Saprolegnia* infection at production scale should be performed.
